# The CAM Model for *CIC-DUX4* Sarcoma and Its Potential Use for Precision Medicine

**DOI:** 10.3390/cells10102613

**Published:** 2021-10-01

**Authors:** Aoi Komatsu, Kotaro Matsumoto, Yuki Yoshimatsu, Yooksil Sin, Arisa Kubota, Tomoki Saito, Ayaka Mizumoto, Shinya Ohashi, Manabu Muto, Rei Noguchi, Tadashi Kondo, Fuyuhiko Tamanoi

**Affiliations:** 1 Institute for Integrated Cell-Material Sciences, Kyoto Universit, Kyoto 606-8501, Japan; komatsu.aoi.6z@kyoto-u.ac.jp (A.K.); matsumoto.kotaro.5r@kyoto-u.ac.jp (K.M.); kubota.arisa.9a@kyoto-u.ac.jp (A.K.); 2 Institute for Advanced Study, Kyoto University, Kyoto 606-8501, Japan; 3 Division of Rare Cancer Research, National Cancer Center Research Institute, 5-1-1 Tsukiji, Chuo-ku, Tokyo 104-0045, Japan; yyoshima@ncc.go.jp (Y.Y.); ishin@ncc.go.jp (Y.S.); renoguch@ncc.go.jp (R.N.); takondo@ncc.go.jp (T.K.); 4 Department of Therapeutic Oncology, Graduate School of Medicine, Kyoto University, Kyoto 606-8501, Japan; tosaito@kuhp.kyoto-u.ac.jp (T.S.); ayaka@kuhp.kyoto-u.ac.jp (A.M.); ohashish@kuhp.kyoto-u.ac.jp (S.O.); mmuto@kuhp.kyoto-u.ac.jp (M.M.)

**Keywords:** *CIC-DUX4* sarcoma, rare cancer, CAM assay, H&E staining, fusion gene, organoids

## Abstract

(1) Background: *CIC-DUX4* sarcoma is a rare mesenchymal small round cell tumor which belongs to rare cancers that occupy a significant percentage of cancer cases as a whole, despite each being rare. Importantly, each rare cancer type has different features, and thus there is a need to develop a model that mimics the features of each of these cancers. We evaluated the idea that the chicken chorioallantoic membrane assay (CAM), a convenient and versatile animal model, can be established for the *CIC-DUX4* sarcoma. (2) Methods: Patient-derived cell lines of *CIC-DUX4* were applied. These cells were transplanted onto the CAM membrane and tumor formation was examined by H&E staining, immunohistochemistry and Western blotting. The CAM tumor was transferred onto a fresh CAM and was also used to form organoids. Retention of the fusion gene was examined. (3) Results: H&E staining as well as molecular characterization demonstrated the formation of the *CIC-DUX4* tumor on the CAM membrane. Expression of cyclin D2 and ETV4 was identified. The CAM tumor was transferred to a fresh CAM to form the second-generation CAM tumor. In addition, we were successful in forming tumor organoids using the CAM tumor. Retention of the fusion gene *CIC-DUX4* in the CAM, second-generation CAM, and in the CAM-derived organoids was confirmed by RT-PCR. (4) Conclusions: The CAM assay provides a promising model for *CIC-DUX4* sarcoma.

## 1. Introduction

*CIC-DUX4* sarcoma (CDS) belongs to a subset of high-grade sarcomas that represents the majority of Ewing-like small round cell sarcomas [1,2,3,4,5]. While Ewing sarcoma harbors a fusion gene that is formed by the fusion of the *EWSR1* gene to the *ETS* gene family [6,7], CDS is characterized by the occurrence of a fusion gene *CIC-DUX4* that is formed by the fusion of *CIC* (Capicua) and *DUX4* (Double homeobox 4 gene). The *CIC* gene encodes a transcription factor with an HMG (high mobility group)-box containing a DNA binding domain [8,9,10]. The *DUX4* gene encodes a double homeodomain transcription activator PITX1 (paired-like homeodomain transcription factor 1) [11,12,13]. The gene expression activated by the *CIC-DUX4* fusion transcription factor differs from that induced by the *EWS* fusion gene [3]. *CIC-DUX4* sarcoma occurs in young patients and optimum treatment options for this tumor need to be established.

Recently, patient-derived xenografts that bear histological similarity with the original patient tumor have been established, and two cell lines have been established from these xenografts [14]. In this study, protein expression profiles were analyzed and the top 10 most enriched pathways have been identified. Another cell line, NCC-CDS2-C1, was established from surgically resected tumor tissue from a CDS patient [15]. These cells exhibited rapid growth, spheroid formation and invasion. The effects of *CIC-DUX4* expression have been investigated and this study showed increased expression of ETV4 and CCNE1 [16]. ETV4 stimulates tumor metastasis, while CCNE controls tumor survival through the CCNE–CDK2 cell cycle complex. More recently, a feedback loop involving DUSP6 (dual specificity phosphatase 6) was identified from the characterization of the *CIC-DUX4* bearing cell line [17,18]. Establishment of these cell lines and xenografts contributed to molecular characterization of the CDS sarcoma.

The CAM (chorioallantoic membrane) assay provides a versatile and powerful tumor model [19,20,21,22,23,24,25,26,27,28,29]. This model uses fertilized chicken eggs that are incubated under constant temperature and humidity with occasional rotation. On days 8–10, a window is opened so that a sample, such as cancer cells or a tumor sample, can be transplanted onto the top of the CAM. We as well as others have shown that a tumor mass bearing excellent resemblance to the original tumor can be formed several days after transplantation. This rapid formation of the tumor in chicken eggs is due to the nutrient-rich environment of the CAM membrane as well as to the incomplete establishment of the immune system in the chicken egg at the time of transplantation. A recent important advance of the CAM assay is that patient tumor samples can be used to transplant on the CAM, enabling the establishment of a patient-derived CAM tumor [24,26,28,29]. 

In this work, we used the patient-derived cell lines of CDS and evaluated the potential for establishing a CDS tumor on the CAM membrane. By using H&E staining as well as by molecular analysis, we show that robust growth of a CDS tumor can be generated that exhibits features similar to those found in the original CDS tumor. In addition, ETV4 expression is demonstrated. The CAM tumor can be transferred to a fresh CAM or used to form CAM-derived tumor organoids. Finally, we show that the *CIC-DUX4* fusion gene is retained in the CAM tumor.

## 2. Materials and Methods

### 2.1. Cells and Media

CD-292 (NCC-CDS2-C1), CD-89A (NCC-CDS1-X1-C1), and CD-89C (NCC-CDS1-X3-C1) sarcoma cell lines used in this study (Supplementary Materials Table S1) were established at the National Cancer Center Japan [14,15]. CD-292 cells were cultured in DMEM/F12 (Gibco, Grand Island, NY, USA) supplemented with 10% heat-inactivated fetal bovine serum (Gibco, Grand island, NY, USA) and 1% penicillin/streptomycin (nacalai tesque, Kyoto, Japan). CD-89A and CD-89C cells were maintained in RPMI-1640 medium (nacalai tesque, Kyoto, Japan) supplemented with the same conditions as DMEM/F12 medium. Cells were incubated at 37 °C and 5% CO_2_. 

### 2.2. CAM Assay

Fertilized white chicken eggs were purchased from Goto farm, Gifu, Japan or Nihon layer, Gifu, Japan. Eggs were incubated in a rotary incubator at 37.5 °C and 65% humidity. Eight-day-old fertilized chicken eggs were used for the CAM to transplant cancer cell lines. After cleaning the egg with 70% ethanol, the air sac of the blunt end was slit to prepare for opening a window on the eggshell. The CAM was dropped by making the window. The window was covered with Opsite film (Smith & Nephew, Watford, London, UK) to avoid the drying until transplanting cells. 

For transplantation, a sterile Teflon ring was placed at the Y-shape blood vessel on the CAM. Then, 2 × 10^6^ cells were grafted into the ring and then the window was covered with Tegaderm film (3M Phoenix Limited, St. Paul, MN, USA). Tumor formation was observed by Olympus SZX12 stereomicroscope on days 3, 5, 7 and 10 after transplantation. To passage CAM tumors, the CAM tumors developed over 8 days after transplantation were cut out, and then grafted onto fresh CAMs which were incubated for 8 days. After grafting, the passaged CAM tumors were observed for 10 days until their cutting out for observation by stereomicroscope on days 3, 5, 7 and 10. CAM tumors were fixed with 4% paraformaldehyde overnight and H&E staining was conducted by Kyoto Institute of Nutrition & Pathology, Inc. (Kyoto, Japan).

### 2.3. Immunohistochemistry

The tumor samples were embedded with paraffin and then cut into sections (3–5 µm thickness). For immunohistochemistry, the sections were subjected to heat-induced epitope retrieval method. The primary antibodies used were: Cyclin D2 (CCND2), 1:200, Abcam, Cambridge, UK; C1 antibody, used as a fibroblast marker, 1:20, DSHB, Iowa, USA; ETV4, 1:50, Proteintech Group, Inc., IL, USA. The sections were observed by Keyence BZ-9000 and BZ-X810 fluorescent microscopes to evaluate tumor development in the CAM.

### 2.4. Immunoblotting

Cells and CAM tumors were lysed in a 2× Cell lysis buffer (Cell signaling technology, Danvers, MA, USA) and then an equal amount of 2× sample buffer was added. CAM tumor was homogenized by Power masherII (Nippi, Inc., Tokyo, Japan) before adding the lysis buffer. After sonication, the lysate was denatured at 100 °C for 5 min. Target proteins were separated and detected with the Jess^TM^ Simple Western system (Protein Simple, San Jose, CA, USA).

### 2.5. RNA Isolation and RT-PCR

Total RNA was extracted from cultured cells and CAM tumors with miRNeasy Mini Kit (Qiagen, Venlo, Limburg, The Netherlands). Itwas then used for reverse transcription with the Superscript III reverse transcriptase (Invitrogen, Carlsbad, CA, USA) according to the manufacturer’s instructions. The *CIC-DUX4* fusion transcript was amplified with the forward primer for *CIC* and the reverse primer for *DUX4*, using Platinum Taq DNA Polymerase High Fidelity (Thermo Fisher Scientific, MA, USA) or KOD-Plus-Neo DNA polymerase (Toyobo, Osaka, Japan). Primer sequence and enzyme for each sample are described in Table S2 of the Supplementary Material. Sanger sequence analysis was performed with the identical primer set for each sample using BigDye v3.1 Cycle Sequencing Kit. The sequence analysis was conducted using the Applied Biosystems 3130xL by GENEWIZ (GENEWIZ, South Plainfield, NJ, USA). The sequence data were matched against the *CIC* sequence (NCBI Reference Sequence: NM_015125.4) and *DUX4* sequence (NCBI Reference Sequence: NM_001293798.2).

### 2.6. Formation of Tumor Organoids from CAM Tumor

CAM tumors established by transplanting CD-292 cells were cut out and were broken into pieces by passing through a 500 µm stainless-steel filter. Enzymatic digestion with Trypsin-EDTA (nacalai tesque) and Liberase (Roche, Basel, Switzerland) was carried out, and the digests were then filtrated using 100 µm cell strainer. Cells were inoculated at 5 × 10^4^ cells per well to a 96 well-U-bottom plate and were incubated in a CO_2_ incubator. Organoid formation was initiated within a week and continued. The medium used was DMEM/F12 for organoid formation.

## 3. Results

### 3.1. Formation of CIC-DUX4 Tumor on the CAM Membrane

Three different cell lines have been established from CIC-DUX4 patients (Supplementary Materials Table S1) [14,15]. The CD-89A (NCC-CDS-X1-C1) and CD-89C (NCC-CDS-X3-C1) cell lines were derived from a 29-year-old female patient, while the CD-292 (NCC-CDS2-C1) cell line was established by using a tumor sample from a 50-year-old female patient. These cells (2 × 10^6^ cells) were used to form CAM tumors on the CAM membrane of fertilized eggs. The fertilized eggs were incubated at 37.5 °C and at 65% humidity for eight days, a window was made, and the cells were placed on top of the CAM membrane as described in Materials and Methods.

**Figure 1:**
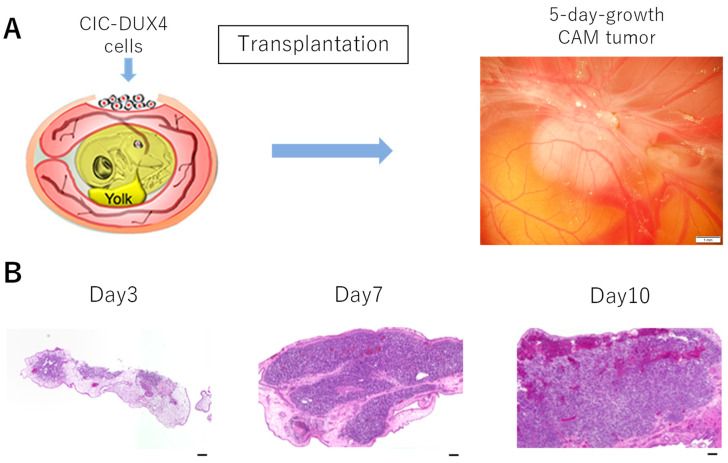
(**A**): After transplanting CD-292 cells on top of the CAM membrane of fertilized eggs, we observed formation of a tumor-like structure seen by white area. Scale bar in the right photo represents 1 mm size. (**B**): The CAM tumor was cut out at different times, and thin sections were made and examined by H&E staining. Scale bar represents 0.1 mm.

Figure 1 shows the results with CD-292 cells. After eight days, a tumor-like formation was observed (Figure 1A). Chronological changes in the formation of tumor are described. As shown in Figure 1B, H&E staining revealed that the tumor was formed on part of the CAM membrane by day 3. This dark stained region reminiscent of a tumor expanded by day 7 and was occupying an entire area. The efficiency of CAM tumor formation was higher than 75%.

**Figure 2:**
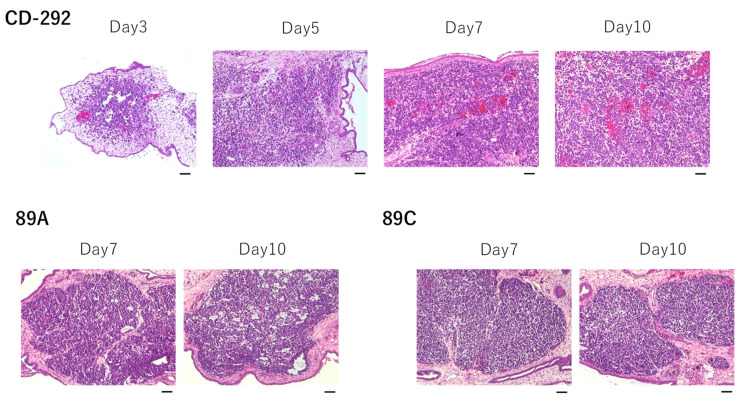
H&E staining of CAM tumors established by transplanting CD-292, 89A or 89C cells. Scale bar represents 0.05 mm.

We present a higher magnification H&E staining of the CAM tumor in Figure 2. A distinctive small round cell morphology was observed, which is similar to the histology of CIC-DUX4 sarcoma published by Choi et al. [30]. Occasionally, a bright red color was observed that represents bleeding, suggesting that tumor blood vessels formed. CAM tumors were also formed by using 89A and 89C cells.

### 3.2. Molecular Features and Tumor Microenvironment

**Figure 3:**
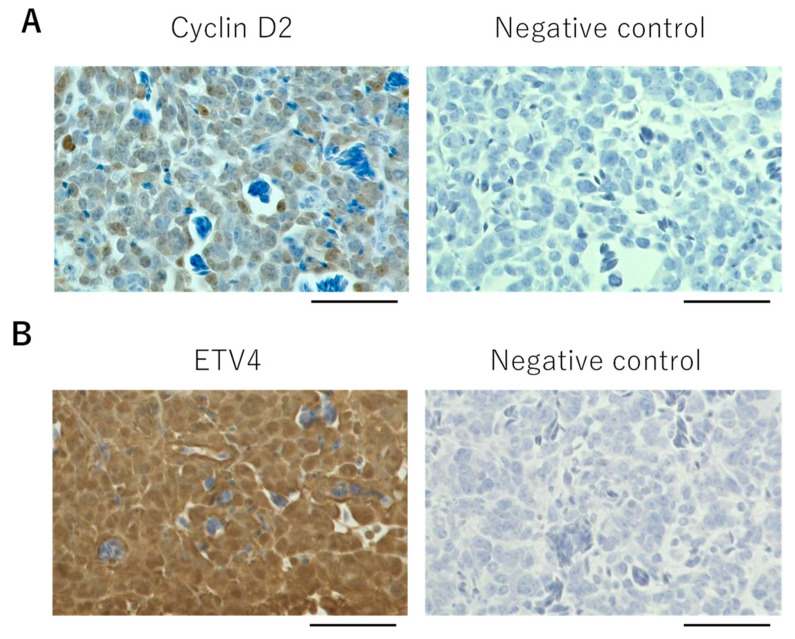
Immunohistochemistry of CD-292 CAM tumor. Stained with cyclin D2 antibody (**A**) or with ETV4 antibody (**B**). Negative controls are staining without antibody. Scale bar represents 0.05 mm.

The *CIC-DUX4* sarcoma (CDS) belongs to a subset of small round cell sarcomas that resemble the Ewing sarcoma (ES) morphology. One of the molecular markers that distinguishes CDS from ES is CCND2 (cyclin D2), which is expressed in CDS but not in ES [31]. Furthermore, gene silencing of Ccnd2 inhibits tumor growth in mice, pointing to the significance of this gene for CDS [31]. Thus, we examined cyclin D2 expression in the CAM tumor. As shown in Figure 3A, staining of the CAM tumor with human cyclin D2 antibody detected a strong signal compared to the negative control. 

**Figure 4:**
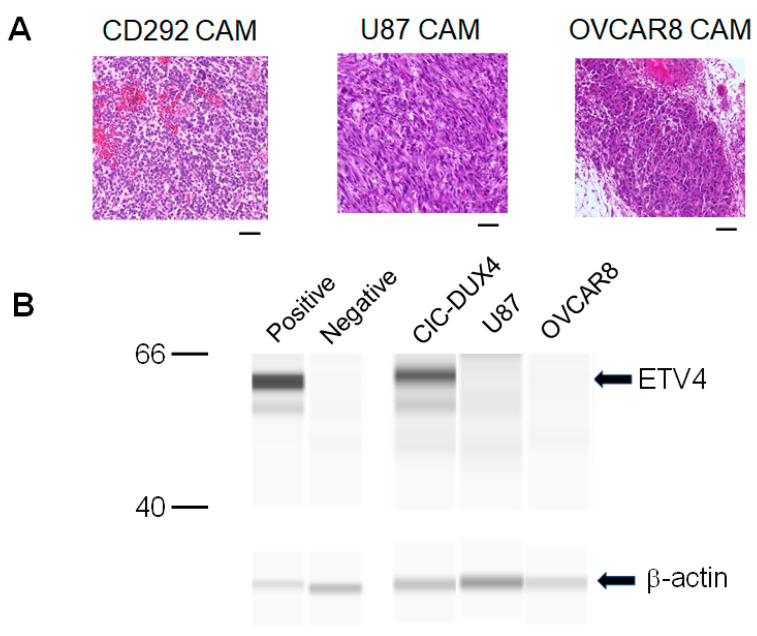
Specific expression of ETV4 in CIC-DUX4 CAM. (**A**): H&E staining of CAM tumors formed by transplanting CD-292 cells (CIC-DUX4 sarcoma), OVCAR8 cells (ovarian cancer) and U87 (glioblastoma). Scale bar represents 0.05 mm. (**B**): Western blot analysis of ETV4 with CIC-DUX4, OVCAR8 and U87 CAM samples. Loading control is β-actin which is shown in the lower panel. Proteins extracted from CAM membrane were used as a negative control, while proteins extracted from CD-292 cells were used as a positive control.

Another gene that distinguishes CDS from ES is *ETV4*. This gene is one of the *PEA3* family genes whose expression is markedly enhanced by the expression of the *CIC-DUX4* gene. Recent immunohistochemical analysis of the *ETV4* gene shows the gene to be a useful marker for the detection of sarcomas with CIC rearrangement [32,33,34]. Immunohistology of the CAM tumor using an antibody against ETV4 exhibited staining of ETV4 (Figure 3B). ETV4 was also detected by carrying out Western blot analysis as shown below in Figure 4. We also identified staining of fibroblast in the CAM tumor using antibodies against chicken proteins (Supplementary Materials Figure S1). Thus, the CAM tumor not only contains CIC-DUX4 cells but also contains chicken fibroblasts that infiltrate into the tumor. As discussed before, we also observed blood vessels formed in the tumor. These results suggest that a tumor microenvironment is formed in the CAM tumor.

We further carried out experiments with ETV4. We prepared two other CAM tumors: an OVCAR8 CAM tumor established by transplanting ovarian cancer cells, OVCAR8-GFP, and a brain tumor established by transplanting U87-GFP cells (Figure 4A). These CAM tumors as well as the CD-292 CAM tumor were collected and lysed, and the presence of ETV4 was examined by carrying out Western blot analysis using antibody against human ETV4 protein. As can be seen in Figure 4B, a band of ETV4 was detected with the CD-292 CAM tumor but not with OVCAR8 or U87 CAM tumors. 

### 3.3. CAM Tumor Can Be Transferred to a Fresh CAM

**Figure 5:**
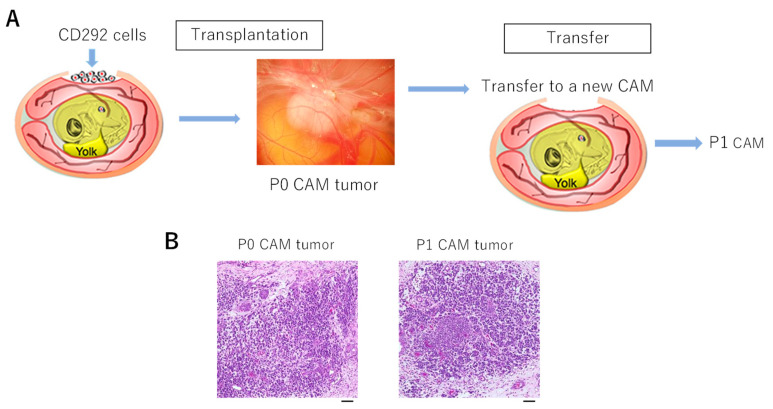
Passaging of CD-292 CAM tumor. (**A**): CD-292 cells were transplanted onto the CAM membrane to produce CAM tumor. This was cut out and minced into small portions and then transplanted onto a fresh chicken egg CAM membrane. (**B**): H&E staining of the passaged CAM tumor was compared with that of the original CAM tumor. Scale bar represents 0.05 mm.

Successful formation of CAM tumor for CD-292 encouraged us to test whether the CAM tumor could be passaged. To test this idea, the CAM tumor formed 8 days after transplantation was cut out and was then transplanted onto a fresh fertilized egg and the incubation continued (Figure 5A). A new tumor (passaged tumor P1) was formed and this was confirmed by H&E staining (Figure 5B). 

### 3.4. Formation of Tumor Organoids from the CAM Tumor

**Figure 6:**
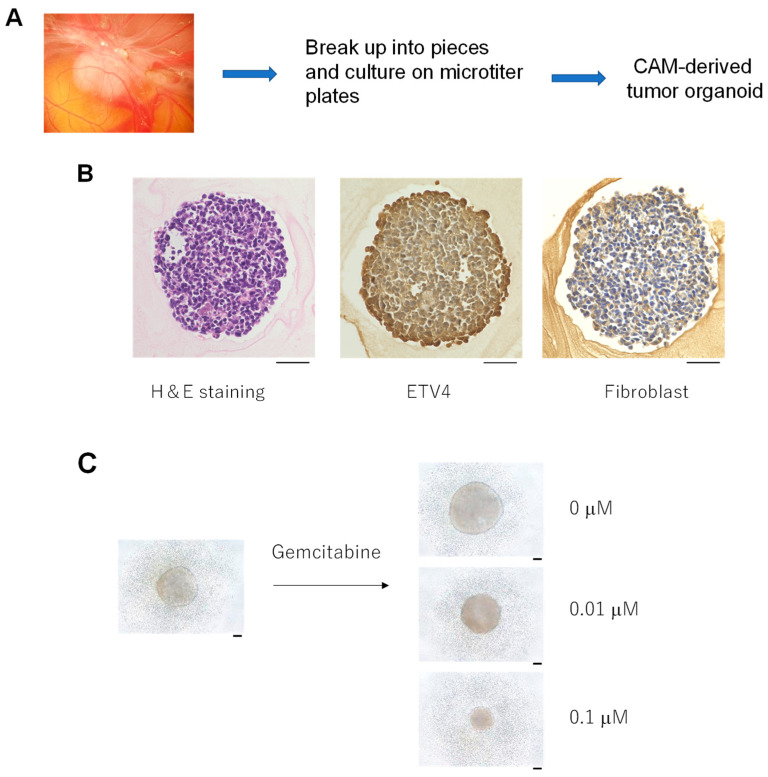
(**A**): CAM tumors can be broken up into pieces by physical and enzymatic methods. They can be cultured to form uniform sized organoids. (**B**): H&E staining and detection of ETV and fibroblasts. Scale bar represents 0.05 mm. (**C**): Sensitivity of CAM organoids to gemcitabine. Test was performed with the tumor organoids. Scale bar represents 0.1 mm.

We found that the CAM tumor formed in the chicken egg can be broken up and cultured on a microtiter plate. The CAM tumors were cut out and then treated with an enzyme to destroy the tumor tissue. The samples were then filtered through meshes of different size to yield near-homogeneous size preparations. They were then placed in a microtiter plate and culture media were added. After incubation for a week, we started to observe three-dimensional aggregates that continued to grow for about 14 days (Figure 6A). These aggregates grew to similar sizes and had similar appearances (Figure 6B). H&E staining is shown in Figure 6B. We also identified ETV4 expression in the CAM-derived tumor organoids (Figure 6B). The characterization of these organoids revealed the presence of chicken fibroblasts (Figure 6B). Thus, we named these structures “CAM-derived tumor organoids”.

If we used five CAM tumors, we could obtain approximately 200 tumor organoids. As a first step towards using these organoids for drug sensitivity tests, we treated them with two different concentrations of gemcitabine and the growth of the organoid was examined. As shown in Figure 6C, the size of the organoids was decreased in a dose-dependent manner in a week, while the size increased in the absence of gemcitabine (PBS control). 

### 3.5. The CIC-DUX4 Gene Is Retained in the CAM, Passaged CAM and CAM-Derived Tumor Organoids

**Figure 7:**
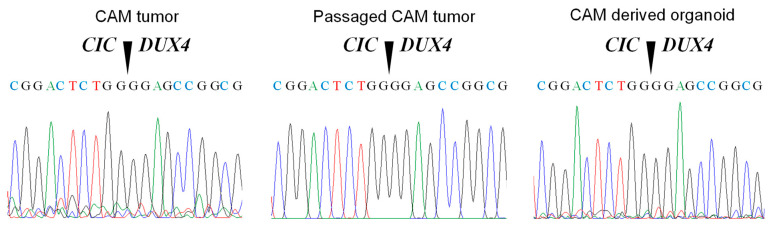
Detection of the CIC-DUX4 gene in the CAM tumor, passaged CAM tumor and in the CAM-derived tumor organoids.

The CD-292 cells express the *CIC-DUX4* fusion gene, which provides a convenient way to assess the CAM tumor as well as CAM-derived tumor organoids. To examine the retention of the *CIC-DUX4* gene in the CAM tumor, we isolated RNA from the CAM tumor and then examined the presence of the fusion gene after an RT-PCR reaction. As can be seen in Figure 7, the fusion gene was detected in the CAM tumor with a sequence identical to that found in the CIC-DUX4-derived cells. The same sequence was detected in the CAM tumor P1 after passaging the primary tumor (P0). Furthermore, we detected the fusion gene in the CAM-derived organoids. Thus, the fusion gene is retained in the CAM tumor as well as in CAM-derived organoids.

## 4. Discussion

Our study described in this paper shows that it is possible to establish a CAM model for the CIC-DUX4 sarcoma. After transplanting patient-derived cells onto the CAM membrane of fertilized eggs, we observed the formation of a tumor-like structure which was then confirmed by H&E staining. The staining revealed the presence of small round cells reminiscent of the CIC-DUX4 sarcoma. Molecular characterization of the CAM tumor employing markers such as cyclin D2, ETV4 or WT1 could thus be carried out [16]. In our study, we showed cyclin D2 expression in the CAM tumor. We also identified the expression of ETV4, a PEA3 family transcription factor that is expressed downstream of the CIC-DUX4 gene [16]. Importantly, the expression of ETV4 was specific to the CIC-DUX4 CAM tumor, as ETV4 was not expressed in other types of CAM tumors derived by transplanting ovarian cancer cells or brain cancer cells. 

In this work, we showed that the CAM tumor formed by transplanting CIC-DUX4 cells can be transferred to a fresh CAM. Furthermore, we found that the CAM tumor can be broken up by enzymatic and separation methods to yield tumor organoids. We examined the presence of the fusion gene CIC-DUX4 in the CAM tumor and passaged the CAM tumor and the CAM-derived tumor organoids. The fusion gene was detected in all these samples, providing convincing evidence that they retain features of the CIC-DUX4 sarcoma. Thus, examining the retention of this gene provides a powerful method to characterize CAM and CAM-derived samples.

In addition, H&E staining as well as immunohistochemistry were carried out with the CAM-derived tumor organoids. This study confirmed the expression of ETV4 and the presence of fibroblasts. The tumor organoids had similar size and structure. From five CAM tumors, we obtained approximately 200 tumor organoids. This provides a unique library of CIC-DUX4-derived organoids that can be used to screen for effective anticancer drugs against the CIC-DUX4 sarcoma. As a first step to evaluate this possibility, we examined gemcitabine sensitivity. We observed a dose-dependent effect on the size of tumor spheroids. Thus, various drugs can be tested for drug sensitivity assays. It will be interesting to test sensitivity to drugs that could affect key players in the signal transduction of the CIC-DUX4 sarcoma. These key events include expression of the CIC-DUX4 gene, cyclin-dependent kinases that control cell cycle [16,31] and DUSP6, a dual specificity phosphatase that is involved in the regulation of ERK phosphorylation to sustain the expression of the CIC-DUX4 gene [17,18].

Rare cancers such as the CIC-DUX4 sarcoma represent an important class of cancer. While each rare cancer has a low incidence, as a whole they represent one of the major classes of cancer cases. However, since each cancer type is different, treatment has to be tailored towards each type of rare cancer [35]. The CAM model provides a valuable approach for meeting this challenge. CAM tumors formed by transplanting rare cancer can be used to carry out molecular characterization. Furthermore, CAM-derived tumor organoids can be used to identify optimum drugs for each rare cancer. Further development of the CAM model for rare cancer is warranted.

## Data Availability

The data presented in this study are contained within the article or the Supplementary Materials.

## Supplementary Materials

The following are available online at https://www.mdpi.com/article/10.3390/cells10102613/s1, Supplementary Table S1: CD292 cell lines used in this study. Supplementary Table S2: Primers used for the detection of the CIC-DUX4 gene. Supplementary Figure S1: CAM tumor derived from *CIC-DUX4* sarcoma cells was stained for the presence of fibroblast using C1 antibody as described in Materials and Methods. Negative control shows staining without the antibody. A scale bar represents 0.05 mm.
