# Supplementary material for: The CAM Model for CIC-DUX4 Sarcoma and Its Potential Use for Precision Medicine
Source: Cells. 2021 Oct 1;10(10):2613. doi: 10.3390/cells10102613 (PMC8533847; doi:10.3390/cells10102613)
Supplement: Supplementary file 1 [file cells-10-02613-s001.zip › cells-1391752-supplementary.pdf]

Supplementary Material

The CAM model for *CIC-DUX4* sarcoma and its potential use for precision medicine

Aoi Komatsu, Kotaro Matsumoto, Yuki Yoshimatsu, Yooksil Sin, Arisa Kubota, Tomoki Saito, Ayaka Mizumoto, Shinya Ohashi, Manabu Muto, Rei Noguchi, **Tadashi Kondo and Fuyuhiko Tamanoi**

Supplementary Table S1: CD292 cell lines used in this study

Cell line label	Patient records
CD-89A (NCC-CDS1-X1-C1)	29-year-old, female
CD-89C (NCC-CDS1-X3-C1)	29-year-old, female
CD-292 (NCC-CDS2-C1)	50-year-old, female

Supplementary Table S2: Primers used for the detection of the *CIC-DUX4* gene

#	Sample name	Forward Primer Sequence (5'-----------3')	Reverse Primer Sequence (5'-----------3')	Enzyme
1	CAM tumor #3	ATCATGCAGGCTGCCACT	ATGCCTTGCATCTGCCC	Platinum Taq DNA Polymerase High Fidelity
2	CAM tumor #4	ATCATGCAGGCTGCCACT	ATGCCTTGCATCTGCCC	Platinum Taq DNA Polymerase High Fidelity
3	CD-292 cell line	ATCATGCAGGCTGCCACT	ATGCCTTGCATCTGCCC	Platinum Taq DNA Polymerase High Fidelity
4	Only CAM	ATCATGCAGGCTGCCACT	ATGCCTTGCATCTGCCC	Platinum Taq DNA Polymerase High Fidelity
5	CAM organoid	ATCATGCAGGCTGCCACT	ATGCCTTGCATCTGCCC	KOD-Plus-Neo DNA polymerase
6	CD-292 TP-10 (Passage tumor)	GGGGACATCTTCACCTTTGA	CCAGGAAAGAATGGCAGTTC	Platinum Taq DNA Polymerase High Fidelity

Supplementary Figure S1

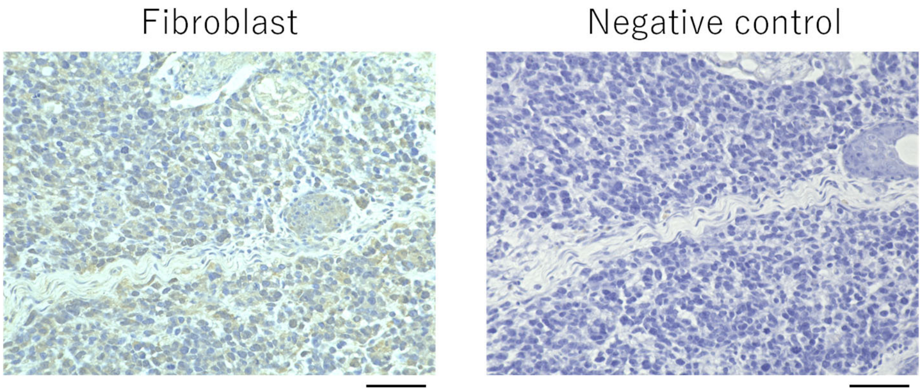

CAM tumor derived from CIC-DUX4 sarcoma cells was stained for the presence of fibroblast using C1 antibody as described in Materials and Methods. Negative control shows staining without the antibody. A scale bar represents 0.05 mm.
